# Effect of Platelet-Rich Plasma on Intervertebral Disc Degeneration *In Vivo* and *In Vitro*: A Critical Review

**DOI:** 10.1155/2020/8893819

**Published:** 2020-11-21

**Authors:** Yvang Chang, Ming Yang, Song Ke, Yu Zhang, Gang Xu, Zhonghai Li

**Affiliations:** ^1^Department of Orthopaedics, First Affiliated Hospital of Dalian Medical University, Dalian, China; ^2^Key Laboratory of Molecular Mechanism for Repair and Remodeling of Orthopaedic Diseases, Liaoning Province, China

## Abstract

Intervertebral disc degeneration (IDD) is a globally occurring disease that represents a significant cause of socioeconomic problems. Currently, the main method for treating IDD is surgery, including discectomy and vertebral fusion. Several *in vitro* experiments demonstrated that platelet-rich plasma (PRP) could stimulate cell proliferation and extracellular matrix regeneration. Additionally, *in vivo* experiments have proven that PRP injection could restore intervertebral disc height. Clinical studies demonstrated that PRP injection could significantly relieve patient pain. However, further studies are still required to clarify the roles of PRP in IDD prevention and treatment. This review is aimed at summarizing and critically analyzing the current evidence regarding IDD treatment with PRP.

## 1. Introduction

Intervertebral disc degeneration (IDD) is becoming a serious medical and social problem. The global prevalence of IDD exceeds 60% and is higher in men and older adults [1], which results in high costs for society. During progress of IDD, dehydration of the nucleus pulposus, annulus fibrosus tears, fracture of the cartilage endplate, and release of inflammatory factors occur. IDD then leads to lumbar disc herniation and discogenic pain, which cause low back pain, and may severely affect spinal extension and flexion. Because of the avascular tissue structure of the intervertebral disc (IVD) with a poor self-healing capacity, oral medication is generally ineffective. Current IDD treatment methods mainly include bed rest, physical therapy, epidural injections, surgical decompression, disc replacement, and disc fusion [2]. Additionally, bioactive agents have attracted considerable attention because of the limited damage caused in their application, in which the basic principle of IDD treatment is to delay or even reverse the trend of IDD through the activity of the agents' components.

Many bioactive agents are currently applied to treat IDD, with most direct treatment strategies for reversing IDD involving the injection of active agents. Platelet-rich plasma (PRP) is particularly attractive because of the high concentration of platelets that releases a variety of multifunctional growth factors (GFs) when activated and has been widely used to repair various avascular tissues, including but not limited to bones, cartilages, muscles, and tendons, and may represent a new strategy for IDD biotherapy [3, 4].

## 2. IVD and IDD

Anatomically, the IVD comprises a central highly hydrated nucleus pulposus (NP), a peripheral thin layer of the annulus fibrosus (AF), and upper and lower cartilage endplates (CEPs) [5]. Histologically, the NP mainly comprises water and a low density of notochord and NP cells and the extracellular matrix (ECM). The ECM mainly comprises collagen II (COL II), elastin, fibers, and numerous proteoglycans especially aggrecan (ACAN) with negative charge. Physiologically, the hydrated NP can absorb the compressive pressure of the spine. The AF mainly comprises COL I as a lamellar ring structure of approximately 15–25 layers outside the NP, and the outer AF layer comprises fibroblasts with a neurovascular distribution, in which the nerves comprise spinal sensory and postganglionic sympathetic fibers [6]. The CEPs are located at both sides of the IVD and comprise a layer of horizontal hyaline cartilage, where nutrients and oxygen enter the IVD by free diffusion.

IVD homeostasis is a complex pathway, in which the factors associated with IDD are mainly genetic and environmental. The genes associated with IDD mainly include three types: (1) genes affecting intervertebral disc structure (ACAN, COL, etc.), (2) genes of metabolic enzymes affecting IVD metabolism (including matrix metalloproteinases [MMPs], cyclooxygenase- [COX-] 2, and tissue inhibitor of matrix metalloproteinases), and (3) inflammation-related genes (including interleukin- [IL-] 1 and IL-6). Changes in expression of these genes may affect homeostasis of the IVD. Furthermore, environmental factors, including repeated mechanical stimulation, poor nutritional supply, deficiencies of oestrogen, and other definite risk factors including obesity, cigarette smoking, and atherosclerosis may affect homeostasis of IVD, which could contribute to IDD [7]. In the early stages of IDD, the content of ACAN and other proteoglycans initially falls. This decrease leads to NP dehydration and atrophy, which alters the NP biomechanics. In advanced cases, the AF will directly bear the longitudinal pressure. The structure of the AF means that it is more resistant to tension than compressive pressure, resulting in a decrease in intervertebral disc height. At the same time, anabolism and catabolic homeostasis of the matrix are disrupted. This process is accompanied by cellular senescence and morphological changes associated with the aging of cells, reduced secretion of matrix proteins, and possible secretion of inflammatory factors. This could then lead to an inflammatory reaction, which further exacerbates progression of IDD. MMP-1, MMP-3, and MMP-13, a disintegrin-like and metalloproteinase with thrombospondin motifs (ADAMTSs), and enzymes that promote catabolism are highly expressed in the degenerated disc. Additionally, COX-2, which may be the cause of pain, is highly expressed. Pigment accumulation in the NP makes it more difficult to distinguish it from the AF, and the NP cell density is simultaneously reduced. These degenerative changes directly lead to a weakening of the mechanical function of the IVD, which in turn leads to structural deformation, disc height loss of the IVD, segmental instability of the spine, and ultimately structural damages, including the AF tearing and NP protrusion, resulting in discogenic pain [8] .

Reactive oxygen species (ROS) are a family of unstable and highly reactive molecules with or without free radicals. ROS are inevitably produced through the oxygen-using metabolic processes of IVD cells residing in an environment of low oxygen tension. Excessive ROS cause oxidative stress, which activates various signaling pathways in disc cells, including the NF-*κ*B pathway, deteriorating the viability and function of disc cells [9]. Injury to IVDs can also cause inflammation-driven ROS production and result in neovascularization and exposure of the otherwise hypoxic resident cells to higher oxygen tension [10].

In summary, oxidative stress induces damage of microenvironmental homeostasis and promotes degradation of the matrix in IVDs. Consequently, a significant loss of elasticity and the normal structure and biomechanical properties of the disc matrix causes IDD.

## 3. PRP

PRP is an autologous blood concentrate that can release abundant *α* particles containing high concentrations of proliferation- and differentiation-promoting GFs and has been widely used in oral surgery and orthopedics [11]. Different equipment and preparation methods may lead to different cells and types of cytokines and their concentrations in PRP. Dohan Ehrenfest et al. [12] defined PRP in four categories according to the content of leukocytes and fibrins: pure PRP (P-PRP), leukocyte-rich PRP (L-PRP), leukocyte platelet-rich fibrin, and pure platelet-rich fibrin. Because PRP is one component of the autologous plasma, it has the following advantages: (1) in principle, immune rejection does not occur; (2) it avoids the spread of diseases; and (3) it can be readily prepared by centrifugation, which is much cheaper than recombinant GFs. Furthermore, the main components of PRP are GFs, inflammatory cells, and adhesion molecules. GFs mainly include platelet-derived growth factor, transforming growth factor-*β* (TGF-*β*), vascular endothelial growth factor (VEGF), and epithelial growth factor. The published experimental results have proven that PRP can promote angiogenesis, cell proliferation, and collagen synthesis, thereby repairing damaged tissues, including tendons, ligaments, cartilage, and other avascular tissue with a low self-healing ability. Notably, PRP may be effective in repairing a patient's atrophic multifidus and compressed nerve roots [13–15]. Inflammatory cells in PRP are mainly leukocytes, and L-PRP may promote regeneration of cartilage and has a strong bactericidal effect. Therefore, PRP has promising prospects in the treatment of osteoarthritis, especially via augmented anti-inflammatory effects, antioxidative chondrocyte proliferation, inhibited MMP-1 activity, and calcification of the matrix [15, 16]. Furthermore, studies have shown that adhesive proteins (fibrin, fibronectin, and vitronectin) in PRP, which can serve as a carrier for stem cells and as tissue-engineering scaffolds, play a role in promoting stem cell therapy with good prospects for application [17].

## 4. *In Vitro* Study of the PRP Repair Function

More than 10 years of research on PRP has gradually demonstrated its potential therapeutic effect for IDD. Many *in vitro* studies explored how PRP functioned. In 2006, Akeda et al. [18] applied PRP to intervening porcine IVD cells (NP and AF cells) and reported that PRP soluble releasate had a mild stimulatory effect on IVD cell proliferation and increased the synthesis of proteoglycans and collagen, whereas platelet-poor plasma (PPP) had no effect. Additionally, PRP displayed stronger stimulative effects in the AF compared with the NP. Chen et al. [19] conducted research on the TGF-*β*1 in PRP and designed a concentration gradient of TGF-*β*1 in PRP to stimulate human NP (hNP) cells. The result showed that PRP enhanced matrix gene expression (COL II and ACAN) and proteoglycan accumulation compared with the PPP intervention group and the control group. Additionally, they demonstrated what the optimum PRP concentration was when it contained 1 ng/ml TGF-*β*1. Furthermore, they proved that the PRP's regeneration effects were via the Smad2/3 pathway phosphorylation. Several years later, a further study of Chen et al. [20] considered the entire IVD *ex vivo*. Porcine IVDs were digested using chymopapain. At 2 weeks after the administration of 10% porcine PRP, mesenchymal stem cells (MSCs), or 10% PRP and MSCs, decreased apoptosis was found and NP cell viability was promoted in this *ex vivo* IVD system. COL II and ACAN mRNA expression was significantly upregulated, and ECM synthesis was promoted. Additionally, they demonstrated that PRP could direct MSC differentiation into chondrocytes. Pirvu et al. [21] compared the effects of different PRP and platelet lysate (PL) concentrations on bovine AF cells. They confirmed that both PRP and PL were able to increase cell proliferation and glycosaminoglycan accumulation in monolayer culture systems and maintain the spherical shape of cells in whole-disc organ culture. When using PRP or PL for an *in vivo* defect site, PRP was better than PL for the formation of a stable biological sponge for absorbing and storing some small molecules that regulated tissue regeneration.

In 2016, Yang et al. [22] also considered TGF-*β*1 and tested the effects of PRP originating from rabbits. They found that PRP stimulated COL II, ACAN, and Sox-9 gene expression, suppressed COL X gene expression, and displayed the ability to stimulate NP cell proliferation. Additionally, they found that 2.5% PRP was the optimum concentration for NP cell stimulation, which was inhibited by a TGF-*β*1 inhibitor or a Smad2/3 signaling pathway inhibitor. Inhibiting TGF-*β*1 signaling significantly prevented the NP cellular expression of Smad2/3 and matrix protein. Nikkhoo et al. [23] reported that with PRP, IDD was able to recover its mechanical properties. Studies have shown that PRP could promote the proliferation of bone marrow NP stem cells, producing an optimal effect when applied at 10%. PRP has the ability to induce undifferentiated MSCs to express chondrocyte phenotype markers, including COL II and ACAN, while reducing the expression of stem cell markers. It also plays an important role in the differentiation of adipose-derived stem cells into NP cells [24–27].

Some researchers studied another mechanism for the treatment of IDD with PRP, namely, the anti-inflammatory effect. In 2014, Kim et al. [28] used the combination of IL-1*β* and tumor necrosis factor- (TNF-) *α* on immortalized hNP (ihNP) cells and demonstrated upregulated MMP-3 and COX-2 expression, whereas ACAN and COL II expression was suppressed. Subsequently, they applied PRP, whereby the NP cells recovered the downregulated COL II and ACAN gene expression, reduced the increased MMP-3 and COX-2 gene expression, and suppressed cytokine-induced proinflammatory degrading enzymes and mediators. In 2014, Liu et al. [29] used lipopolysaccharides to induce an inflammatory condition in ihNP cells. There were changes similar to IDD. Sox-9, COL II, and ACAN expression on culture with PRP in two dimensions were strongly upregulated, and the inflammatory factors, including IL-1*β*, TNF-*α*, and MMP-3, were absent. In 2016, Cho et al. [30] applied TNF-*α* to treat porcine AF cells and found that MMP-1 was induced by TNF-*α*. Then, with cocultivation by PRP, they came to a conclusion that PRP upregulated COL II and ACAN gene expression and inhibited MMP-1 expression.

Other opinions have been presented regarding the effects of PRP. High leukocyte concentrations in L-PRP may increase the levels of proinflammatory factors (the major components were IL-1*β* and TNF-*α*) and exacerbate the degradation of ECM. Yin et al. [31] also demonstrated that IL-1*β* and TNF-*α* from L-PRP could activate the NF-*κ*B signaling pathway and promote prostaglandin E2 and nitric oxide production, resulting in inhibited tissue regeneration.

Wang et al. [24] demonstrated that L-PRP treatment of NP-derived stem cells significantly upregulated the expression of the inflammatory genes IL-1*β* and TNF-*α* and promoted catabolism. The authors believed that leukocyte exclusion from PRP might effectively prevent the activation of the NF-*κ*B signaling pathway.

Jia et al. [25] used P-PRP or L-PRP to treat rabbit NP MSCs (NPMSCs) *in vitro*. The results showed that P-PRP decreased the expression of stem cell markers and stimulated differentiation into NP-like cells. Additionally, P-PRP had a dose effect on NPMSCs, with the maximum proliferation at 10%. Because L-PRP has significantly higher TNF-*α* and IL-1*β* concentrations, it induced differentiation of NPMSCs, upregulated TNF-*α* and IL-1*β* expression, enhanced activation of the NF-*κ*B pathway, and increased MMP-1 and MMP-13 expression. Inflammation and catabolic reactions resulted in reduced ECM production in differentiated NPMSCs while P-PRP did not activate the NF-*κ*B pathway.

Conversely, Mietsch et al. [32] put forward different views in 2013. They reported that while PRP administration had stimulatory effects on cell proliferation in cultured NPC and MSC cultures, gene expression of chondrogenic markers (ACAN, COL II, COL I, and Sox-9) was considerably lower compared with TGF-*β*1, and matrix protein deposition was weaker. Therefore, they did not recommend PRP to be applied in hNP tissue-engineering because of a probable lack of an effective activator.

Hondke et al. [33] conducted a study on the proliferation, migration, and ECM formation of early AF cells having Pfirrmann grades 2–3 and advanced AF cells having Pfirrmann grades 4–5 from the degenerative tissue. They reported that 5% PRP was the optimum concentration for cell migration, while COL I expression was decreased at this PRP concentration. Therefore, these authors were unable to conclude whether an AF-like ECM could be formed after stimulation by PRP. In summary, 5% PRP has an optimal chemotactic effect, but COL I expression in AF cells is significantly decreased. The authors believed that PRP treatment inhibits matrix formation of the AF.

In conclusion, *in vitro*, PRP was effective in stimulating IVD cell proliferation and ECM metabolism. The antiapoptotic and anti-inflammatory effects of PRP might contribute to disc repair and symptom relief in IDD patients in an early stage. Some mechanisms for PRP treatment of IDD, including the Smad pathway, were proposed over time and a direction for future research is of PRP promotion of MSC differentiation into NP cells (Table 1).

## 5. *In Vivo* Study on Animal Models Treated with PRP

To further explore the effect of PRP on IDD, numerous *in vivo* studies have been performed.

In 2009 and 2006, Kazuhide et al. and Nagae et al. [34, 35], respectively, used an IDD rabbit model with partial discectomy and found that PRP may inhibit IDD while PRP combined with gelatin gel microspheres could significantly inhibit IDD. Compared with the IDD control group, magnetic resonance imaging (MRI) showed a significant increase in intervertebral disc height on PRP application. Histological examination detected increased ACAN and COL II expression and decreased apoptosis of NP cells. Additionally, the gelatin gel microspheres served as a carrier for PRP, prolonging its action time and providing mechanical support, whereby PRP could fully exert its therapeutic effect.

In 2009, Chen et al. [20] injected PRP into a porcine disc degeneration model induced by chymopapain. After 2 months, COL II and ACAN mRNA expressions were upregulated, MSC differentiation and fewer apoptotic cells were detected, and MRI showed recovery of the disc height index (DHI). GB et al. [36] demonstrated that no matter when PRP was injected in the IDD model, there was a protective effect on IDD. However, earlier administration in the IDD process may be more beneficial than PRP treatment of advanced degenerated discs.

Obata et al. [37] in 2012 found that the administration of active autologous PRP releasate could induce a remedial effect on a rabbit needle puncture model. Compared with the PPP and phosphate-buffered saline groups, autologous PRP induced restoration of the DHI, the mean T2 values in MRI were not significantly different, and the number of chondrocyte-like cells was significantly higher. Their results suggested that PRP injection treatment had the potential for clinical application in IDD. Gui et al. [38] conducted a preclinical study of autologous PRP activated by thrombin and found the same result. Two and four weeks after PRP application, they found an increased DHI and signal intensity and elevated proteoglycan and COL II expression compared with the control group, which suggested that PRP intervention may be able to effectively suppress the IDD process.

Wang et al. [39] injected PRP with bone marrow-derived MSCs (BMSCs) in a rabbit AF puncture model and found that the ECM and cell densities were well preserved and increased T2 signal intensity on MRI grading, while a strong immunopositive staining for COL II showed a poor AF recovery. The MRI scores of the PRP group were similar to those of the PRP+BMSC group at 2 weeks, yet the efficacy of PRP+BMSC was diminished at 8 weeks. They hypothesized that the injected PRP was activated by the surrounding tissues and interacted with BMSCs to cure IDD.

In a rabbit study, Yang et al. [22] reported that MRI revealed a significant recovery of the signal intensity of T2 in the IVD of the PRP injection group compared with the very low signal intensity in the control group. In their histological study, the group of combining PRP and TGF-*β*1 inhibitor displayed significantly lower expression levels of Smad2/3 and COL II than the PRP group. Additionally, inhibiting the TGF-*β*1/Smad2/3 pathway could prevent the PRP-induced recovery by inactivating Smad2/3 and downregulating the ECM. They concluded that the TGF-*β*1/Smad2/3 pathway might play a critical role in the ability of PRP to cure IDD.

Hou et al. [40] investigated the effects of the combined application of bone morphogenetic protein 2 (BMP2) and PRP to BMSCs. They reported that BMSCs transduced with BMP2 in PRP gel inhibited IDD with a relatively well-preserved NP structure and enhanced ECM accumulation in the NP. At 12 weeks after surgery, BMP2-transduced BMSCs could be detected.

The quantitative analysis by Li et al. [41] of previous studies showed that PRP treatment could restore the intervertebral disc height, reduce the degree of histological variation, and effectively increase the MRI T2 signal without a significant increase in COL II expression.

**Figure 1:**
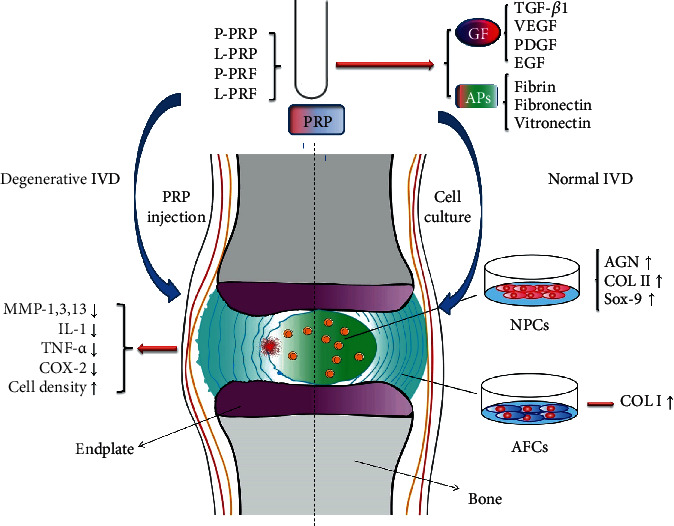
Mechanism of PRP on intervertebral disc cells and degenerated intervertebral disc. PRP: platelet-rich plasma; P-PRP: pure PRP; L-PRP: leukocyte platelet-rich PRP; P-PRF: pure platelet-rich fibrin; L-PRF: leukocyte platelet-rich fibrin; IVD: intervertebral disc; GFs: growth factors; APs: adhesive proteins; MMPs: matrix metalloproteinases; IL: interleukin; TNF: tumor necrosis factor; COX: cyclooxygenase; AGN: aggrecan; COL: collagen; Sox-9: SRY-related high-mobility group box-9; NPCs: nucleus pulposus cells; AFCs: annulus fibrosus cells.

A consensus shows that the injection of PRP or its releasate is effective in enhancing ECM accumulation, retaining a high DHI, increasing the T2 signal intensity, and slowing the process of and even possibly reversing the effects of IDD. Therefore, the releasate may be a promising therapy for retarding IDD. More studies are currently focusing on combining either the PRP injection with other agents or the PRP activation pathways to determine how PRP is effective and to determine limitations as well as ways to optimize this potential treatment. *In vivo* animal studies alone are not sufficient to prove whether PRP injection can alleviate clinical symptoms (Table 2 and Figure 1).

## 6. Clinical Trials of PRP

The forms of PRP injections mainly include intradiscal and epidural, while intradiscal injection is considered first depending on the specific injection position. The following section will review recent studies on clinical PRP injection.

In 2011, Koji et al. [42] conducted the first preliminary clinical trial for the safety and efficacy of intradiscal injection therapy using PRP for IDD. They injected PRP activated by autologous serum and CaCl_2_ in six patients who had suffered chronic low back pain for more than 3 months. After a 1-month follow-up, the scores of the verbal pain scale (VPS) and Roland-Morris Disability Questionnaire were significantly decreased. Additionally, at the 6-month follow-up, MRI did not show any significant change compared with before the injection. No adverse events were reported posttreatment. A few years later, they conducted another preliminary clinical trial including 14 patients with a mean follow-up period of 10 months [43]. In this study, they injected P-PRP isolated by the buffy coat method containing lower concentrations of proinflammatory cytokines (IL-1, TNF-*α*). The mean pain scores of patients before treatment were reduced by more than 70% at the first month. Except for two subjects with recurring low back pain, there were no adverse events. Akeda et al. showed the safety, feasibility, and efficacy of PRP in treatment of IDD.

Bodor et al. [44] injected PRP excluding leukocytes and erythrocytes into 47 thoracic or lumbar IVDs in 35 patients, and no activators were used for the PRP. The numerical rating scales (NRS) and the Oswestry Disability Index (ODI) scores were improved in 67% of the patients. Five patients were followed up for 10 months; these patients displayed substantial improvement in pain, enabling them to return to the normal physical activity. Despite 2 of 35 patients having vasovagal episodes, there were no complications or side effects related to this treatment.

In a case series study by Navani and Hames [45], six patients were given a single injection of autologous PRP. During a 6-month follow-up period, 50% of the VPS scores of all the patients decreased by 3 months, and low pain levels were maintained until the 6-month follow-up. The short-form 36 health survey questionnaire was also improved in both the physical and mental scores with no adverse effects reported.

In 2016, Levi et al. [46] reported a prospective clinical trial of 22 patients investigating the effects of intradiscal L-PRP injections on discogenic back pain. After a 6-month follow-up period, 47% had a successful outcome defined as at least a 50% improvement in the VAS and at least a 30% improvement in the ODI. The authors speculated that the possible adverse effects from using the anesthetics and antibiotics before the 1.5 ml L-PRP injection and the PRP preparation method accounted for positive outcome being nonsignificant in this study.

None of the above studies included a randomized controlled trial (RCT). Tuakli et al. [47] conducted a double-blind RCT of intradiscal PRP therapy for discogenic low back pain (LBP). In total, 47 adults were included with chronic LBP (>6 months), and they were randomly assigned to the treatment group or the control group in a ratio of 2 : 1. The treatment group was given a single injection of L-PRP without an activator. At an 8-week follow-up, they demonstrated that in the treatment group compared with the control group, the NRS for pain, the functional rating index, and patient satisfaction (NASS outcome questionnaire) were significantly improved. In total, 56% (15/27) of the participants were satisfied (NASS outcome questionnaire) with the treatment compared with only 18% (3/17) of the control participants (3 participants were lost to follow-up). However, the results were not compared after 8 weeks because of the lack of a follow-up of the control group. No complications were reported.

In 2017, Lutz [48] presented a single case report demonstrating the effect of intradiscal PRP injection on improving LBP and function. The patient was diagnosed with IDD and had received an ineffective caudal epidural steroid injection and physical therapy. The patient was given a single PRP injection and displayed considerable improvement in pain and motion after 6 weeks. At a 1-year follow-up, there was a remarkable improvement in the LBP, and the patient could return to athletic activities. Additionally, the patient's NP T2 signal intensity increased.

Comella et al. [49] injected PRP combined with stromal vascular fraction, which was enriched in GFs and stem cells extracted from autologous fat tissues. After 6 months of follow-up, the VAS, ODI, and BDI data displayed a positive trend, and a majority of the patients reported improvements in their Dallas Pain Questionnaire scores. No complications were reported.

In conclusion, the results of clinical application of PRP have been shown in many studies. Large-scale, double-blind randomized studies with well-controlled conditions of preparation and sufficient power, as well as effective analyses of PRP components, are required to establish an evidence-based and standardized treatment of IDD with PRP (Table 3).

## 7. Conclusion

In this review, we describe the effects of PRP with a focus on *in vitro* and *in vivo* (animal) studies, which revealed that PRP has significant biological effects in stimulating IVD cells to repair tissues and in intradiscal therapy from basic to clinical research for the treatment of discogenic LBP caused by IDD. The results are quite encouraging, and autologous PRP could be rapidly and readily prepared through a complete set of professional equipment.

The aforementioned experiments and clinical studies define PRP as a promising biological material. Furthermore, the three-dimensional network structure of the PRP gel is beneficial in nutrient acquisition of seed cells and metabolite circulation. PRP gel shows promise as a scaffold material for NP tissue engineering. The PRP gel is endowed with remarkable biomechanical properties and a three-dimensional network structure, which could be used alone as a growth factor source or as a tissue engineering scaffold material combined with seed cells for degenerative disc repair and tissue engineering in NP reconstruction. Although the results provided invaluable insights, they still require further research prior to application. (1) While the mechanism of TGF-*β*1 is well-characterized, how it interacts with other grow factors remains unclear. (2) The action of multiple intradiscal injections may damage the AF, and repeated intradiscal injections might cause an increase in the pressure of the IVD, which may accelerate the IDD process. (3) GFs (including VEGF) contained in PRP may further exacerbate the endogenous neurovasculature of IVD. (4) IDD is frequently associated with ossification of the CEPs with an accompanying decrease in oxygen and nutrients. Moreover, the regeneration of IVD cells is accompanied by various synthetic reactions of ACAN and COL II, which may accumulate lactic acid and accelerate the IDD process. (5) The indication for IDD treatment, the optimal PRP concentration and its composition, and how to evaluate the effect of IVD regeneration require investigation. (6) The role of PRP depends on the number of viable cells, but viable cells available in patients with advanced IDD are extremely limited. (7) The ideal amount of PRP injections and when and what timing clinicians should consider for application of PRP treatment remain unknown. (8) The potential adverse effects of PRP treatment are also unknown. Therefore, combining PRP therapy and stem cell implantation may provide new directions for IDD treatment strategy and IVD repair.

## Figures and Tables

**(a):** 

Year	Study	System used to obtain PRP	Type of cells	Dose
2006	Akeda et al. [18]	SYMPHONY	Porcine IVD NP cell AF cell	10% PRP, 10% PPP
2006	Chen et al. [19]	Centrifuged	hNP	PRP (defined as TGF-*β*1 equivalent)
2009	Chen et al. [20]	Centrifuged	Porcine IVD organ induced with chymopapain	10% porcine PRP
2013	Mietsch et al. [32]	Centrifugation and double filtration	hNP MSC	10% PRP MSC
2014	Kim et al. [28]	GPS III System	hNP	5%, 10% PRP
2014	Liu et al. [29]	Not declared	ihNP	PRP (defined as TGF-*β*1 1 ng/ml equivalent)
2014	Pirvu et al. [21]	INTERCEPT Blood System	Bovine AF cells	25%–50% human PRP 25%–50% human platelet lysate
2016	Yang et al. [22]	Centrifuged	Rabbit NP cells	10%, 5%, 2.5%, and 1% volume fractions of PRP
2016	Cho et al. [30]	Centrifuged	Porcine AF cells with TNF-*α*	PRP of 1, 5, 10 × 10^7^ platelets/ml
2018	Wang et al. [24]	Two-step centrifugation	Rabbit NP-derived stem cells	5%–20% rabbit P-PRP or L-PRP
2018	Jia et al. [25]	Two-step centrifugation	NPMSCs	10% P-PRP or 10% L-PRP
2018	Hondke et al. [33]	Not declared	hAF	PRP 5%

**(b):** 

Time of analysis	Activator	Results
72 h	Thrombin+CaCl_2_	Cell proliferation↑, PG and col synthesis↑, PG accumulation↑
7, 9 days	Thrombin	NP cell proliferation and aggregation↑; optimum at 1 ng TGF-*β*1 concentration in PRP. Tissue construct ↑ COL II, AGN, SOX-9 mRNA ↑, GAG accumulation ↑phosphorylation of Smad2/3 ↑, apoptosis ↓
4 weeks	Thrombin	NP regeneration ↑mRNA involved in chondrogenesis and matrix accumulation↑
7 days, 4 weeks	Acetic acid	Cell proliferation↑ chondrogenic differentiation↓
48 h	CaCl_2_	IL-1*β* and TNF-*α* led matrix synthesis gene expression↓; PRP degradation expression of COX-2 and MMP-3↓
7 days, 4 weeks	None	The expression of chondrogenic markers↑, inflammatory mediators ,matrix degrading enzymes in ihNPc↓
2, 4 days	Sonication	50% PL-DNA and GAG ↑. Matrix synthesis↑ of defect AF after PRP injection
1, 2, 3, 4, 5, 6, and 7 days	10% CaCl_2_ 100 U thrombin	mRNA of COL II, AGN, and SOX-9↑; protein of COL X level TGF-b1/Smad2/3 ↑ COLII and AGN↑ by 2.5% PRP
24 h	1N HCL	COL II and AGN mRNA ↑,MMP1 mRNA, protein ↓
14 days	None	P-PRP: AGN, COL II↑, IL-1*β*, TNF-*α*, IL-6, IL-8, MMP-1, MMP-13 mRNA, IL-1*β*, TNF-*α* production ↓
7 days	10% CaCl_2_	P-PRP: AGN, COL II↑ L-PRP: IL-1*β*, TNF-*α* MMP1, MMP-13 mRNA ↑ NF-*κ*B/p65 protein↑
0, 7, 14, 21 days	Freezing and thawing	Stimulated migration and cell viability in early COL II mRNA ↑, COL1 and 3 mRNA ↓

Abbreviations: ACT: activation; NP: nucleus pulposus; AF: annulus fibrosus; AGN: aggrecan; COL II: type II collagen; COX-2: cyclooxygenase-2; GAG: glycosaminoglycan; IVD: intervertebral disc; MMP: metalloproteinase; MSC: mesenchymal stem cell; Sox-9:SRY-related high mobility group box gene-9; PG: proteoglycan; PRP: platelet-rich plasma; P-PRP: leukocyte-poor PRP; R-PRP: leukocyte-rich PRP; TNF: tumor necrosis factor.

**(a):** 

Year	Study	Animal model	Dose	Size of sample	Time of analysis after injection
2007	Nagae et al. [35]	Rabbits, nucleotomy	20 *μ*l	36	2, 4, and 8 weeks
2009	Kazuhide et al. [34]	Rabbits, nucleotomy	20 *μ*l	128	2, 4, and 8 weeks
2009	Chen et al. [20]	Miniature porcine, chymopapain	No mention	14	4 and 8 weeks
2011	GB et al. [36]	Rats, needle puncture	100 *μ*l	18	2, 4, and 6 weeks
2012	Obata et al. [37]	Rabbits, needle puncture	20 *μ*l	12	8 weeks
2015	Gui et al. [38]	Rabbits, needle puncture	100 *μ*l	36	2 weeks
2016	Wang et al. [39]	Rabbits, needle puncture	200 *μ*l	40	1, 2, and 8 weeks
2016	Yang et al. [22]	Rabbits, needle puncture	15 *μ*l	24	0, 4, 8, and 12 weeks
2016	Hou et al. [40]	Rabbits	40 *μ*l	60	4 and 8 weeks

**(b):** 

Injection site	Activator	Results
Lumbar	None	PRP+GHM group decreased IDD and increased PG; PRP+PBS group showed no differences
Lumbar	None	PRP+GHM had greater DHI, water content, AGN, and COL II mRNA↑; fewer apoptotic cells in NP
Thoracic lumbar	None	PRP promote DHI and osteogenic MSC differentiation
Lumbar	None	Preserved IVD fluid content, decreased IVD degeneration
Lumbar	Autologous serum and 2 % CaCl_2_	Increased cell proliferation, no statistical differences on MRI findings
Lumbar	0.06 ml thrombin	DHI maintained, NP signal intensity maintained; significantly low MRI grading
Lumbar	None	PRP moderate effect on MRI, DHI PRP +BMSC: well-preserved ECM, cell density, increased T2 signal intensity, MRi grading, and expression of COL II
Lumbar	100 U thrombin + 10% CaCl_2_	T2 signal intensity: PRP > control or PRP+TGF-*β* inhibitor; histology: PRP-less degeneration, strong COL II staining, and Smad2/3-pathway activated
Lumbar	Thrombin+CaCl_2_	COL II and PG staining and MRI grade PRP + BMP2 – BMSC > PRP + BMSC > PRP

Abbreviations: ACT: activation; AGN: aggrecan; BMSC: bone marrow-derived mesenchymal stem cell; BMP2: bone morphogenetic protein 2; COL II: type II collagen; ECM: extracellular matrix; GHM: gelatin hydrogel microsphere; IVD: intervertebral disc; MRI: magnetic resonance imaging; PG: proteoglycan; PRP: platelet-rich plasma; DHI: disc height index.

**(a):** 

Year	Study	Study design	Number of patients	Type of PRP	Activation
2011	Koji et al. [42]	Prospective preliminary trial	6	P-PRP releasate	CaCl_2_+autoserum
2014	Bodor et al. [44]	Case series	35	P-PRP	None
2016	Navani and Hames [45]	Case series	6	L-PRP	None
2016	Levi et al. [46]	Prospective trial	22	L-PRP	None
2016	Tuakli-wosornu et al. [47]	Double-blind randomized	36 treatments and 22 controls	L-PRP	None
2017	Akeda et al. [43]	Prospective trial	14	P-PRP	CaCl_2_+autoserum
2018	Lutz [48]	Single case report	1	L-PRP	None

**(b):** 

Volume of whole blood	Volume of PRP injected	Number of injections during the study period	Study period	Pain scores evaluated in the study
200 ml	2 ml	Single	6 m	VAS RDQ
9 ml	2 ml	Single	2-10 m	NRS ODI
60 ml	1.5-3 ml	Single	24 w	VPS
30 or 60 ml	1.5 ml	Single, at one or multiple levels	6 m	VAS ODI
30 ml	1-2 ml	Single, at one or multiple levels	8 w	FRi, NRS, SF-36, and modified NASS
200 ml	2 ml	Single	10 m	VAS RDQ
Not mentioned	1.5 ml	Single	12 m	Improvement T2 nuclear signal intensity↑

Abbreviations: ACT: activation; BDI: Beck Depression Inventory; DPQ: Dallas Pain Questionnaire; FRI: functional rating index; L-PRP: leukocyte- and platelet-rich plasma; m: months; NRS: numerical rating scale; ODI: Oswestry Disability Index; P-PRP: leukocyte-poor PRP; PRP: platelet-rich plasma; RDQ: Roland-Morris Disability Questionnaire; SF: short form; SF-MPQ: short-form McGill Pain Questionnaire; vPS: verbal pain scale; w: weeks; VAS: visual analog scale; NASS: North American Spine Society.

## Data Availability

No data were used to support this study.
